# A coordinated multi-target approach based on chondroitin sulphate nano-selenium mitigates Alzheimer’s disease pathologies in APP/PS1 mice

**DOI:** 10.3389/fphar.2026.1936198

**Published:** 2026-09-15

**Authors:** Dongsheng Ji, Jing Zhao, Xinhui Huang, Xuan Liu, Qirong Yang, Xinfeng Li, Lei Yan, Debo Gao, Yuliang Xiao

**Affiliations:** 1 Shandong Cancer Hospital and Institute, Shandong First Medical University and Shandong Academy of Medical Sciences, Jinan, Shandong, China; 2 Department of Pharmacy, Shandong Provincial Taishan Hospital, Taian, Shandong, China; 3 School of Pharmaceutical Sciences, Shandong First Medical University and Shandong Academy of Medical Sciences, Jinan, Shandong, China; 4 Department of Hematology, Qilu Hospital, Cheeloo College of Medicine, Shandong University, Jinan, Shandong, China

**Keywords:** alzheimer’s disease, chondroitin sulfate nano-selenium, multi-target, neuroinflammation, tau phosphorylation, β-amyloid deposition

## Abstract

Alzheimer’s disease (AD) is a severe neurodegenerative disorder characterised by progressive memory loss and cognitive decline. Because of the lack of effective treatment modalities for AD, novel therapeutics that could delay the progression of AD are urgently required. In this study, the neuroprotective effects and mechanisms of chondroitin sulphate nano-selenium (CS@Se) were investigated by using APPSwe, PSEN1dE9 (APP/PS1) transgenic mice. We found CS@Se to exhibit potent multi-targeted anti-AD effects, and it showed excellent ability to effectively alleviate neuropathy and cognitive dysfunction. Behavioural experiments showed that CS@Se improved learning and memory capacity and enhanced motor activity. Mechanistically, CS@Se upregulated A disintegrin and metalloproteinase domain-containing protein 10 (ADAM10) expression while downregulating the levels of amyloid precursor protein (APP) and beta-secretase 1 (BACE1). Further studies revealed that CS@Se could reduce β-amyloid (Aβ) deposition and inhibit tau phosphorylation by promoting APP hydrolysis and inhibiting the glycogen synthase kinase (GSK)-3β signalling pathway in APP/PS1 mice. Additionally, CS@Se could alleviate oxidative stress damage, neuroinflammation, and cholinergic damage. In conclusion, CS@Se confers substantial neuroprotective effects by modulating several key pathological pathways, providing evidence for its multi-target therapeutic strategy against AD.

## Graphical Abstract

**Figure:**
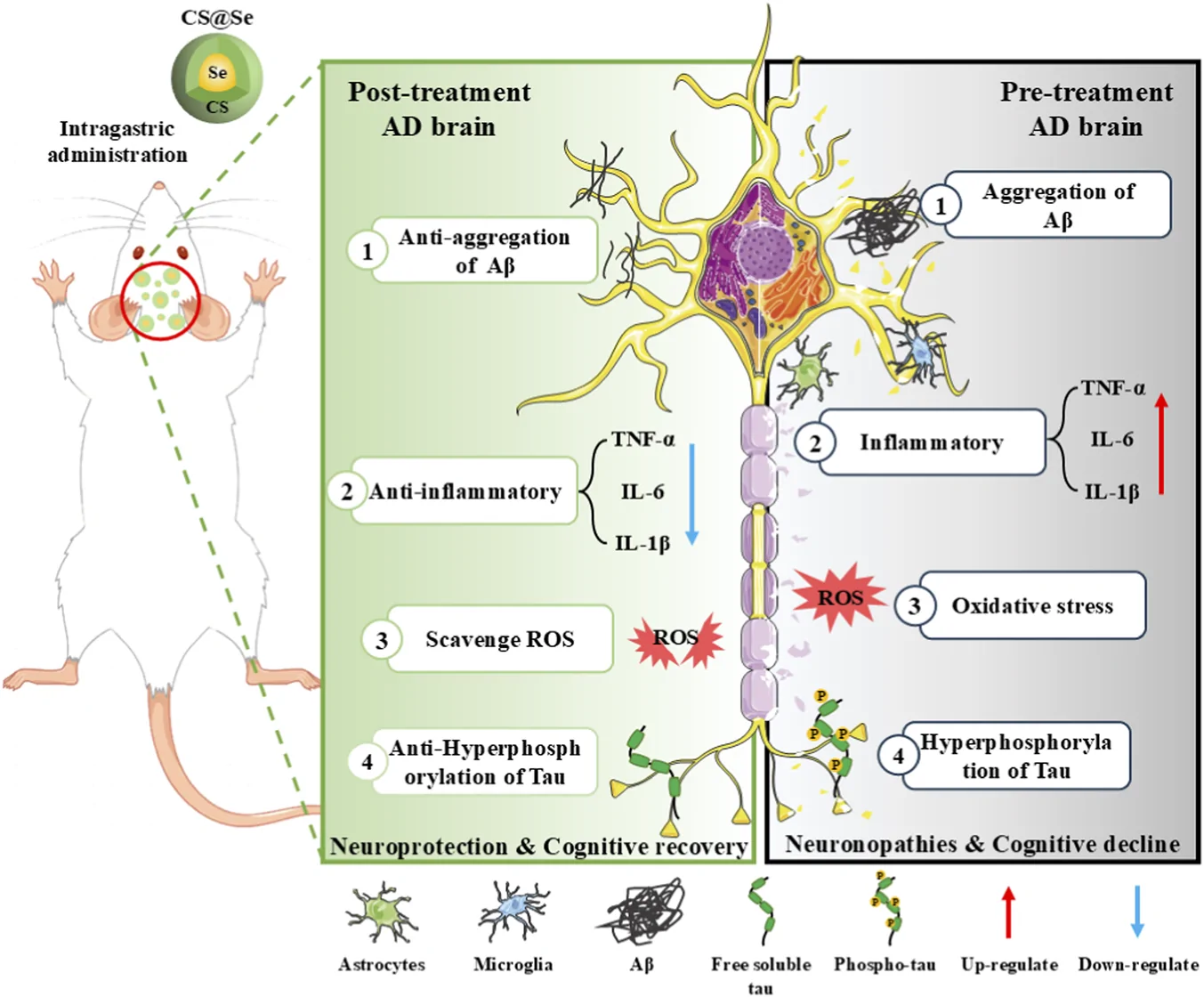

## 1 Introduction

Alzheimer’s disease (AD) is an age-dependent neurodegenerative disease that causes progressive memory impairment and cognitive dysfunction in the elderly. The incidence of AD has been increasing with the ageing of the global population, imposing a substantial burden on families and societies. As a multi-factorial disease, AD has a relatively complex pathogenesis (Guo et al., 2020; Wang et al., 2020). Numerous studies have indicated that abnormal metabolism of amyloid precursor protein (APP) (Hung et al., 2024), excessive tau protein phosphorylation (Harrison et al., 2020), oxidative stress damage (Bai et al., 2022), neuroinflammation (Calsolaro and Edison, 2016), genetic factors (Miller et al., 2022), and environmental factors (Wu et al., 2023) may all play important roles in the pathogenesis of AD. To date, FDA has approved two major categories based on their mechanisms of action for the treatment of AD. The first category comprises symptomatic treatments, including acetylcholinesterase inhibitors (such as donepezil) (Marucci et al., 2021), *N*-methyl-D-aspartate (NMDA) receptor antagonists (such as memantine) (Tariot et al., 2004), which provide modest symptomatic relief. The second category consists of disease-modifying therapies, represented by Aβ-directed monoclonal antibodies (such as Aduhelm and Lecanemab) (Cummings, 2023; Dhillon, 2021). These antibody therapies represent approved disease-modifying therapies. Although their clinical impact remains modest, their intended mechanism extends beyond symptomatic management. However, these traditional single-target drugs can only provide limited relief for certain symptoms. They neither halt disease progression nor cure AD. Therefore, given the complex pathogenesis of AD and the therapeutic limitations of single-target drugs, identification and development of multi-target interventional drugs with synergistic effects may provide novel solutions.

Chondroitin sulphate (CS), a sulphated glycosaminoglycan widely present in animal cartilage tissue (Yang et al., 2024), shows notable pharmacological activity, including neuroprotective effects (Medeiros et al., 2023), antioxidative properties (Ou et al., 2023), as well as anti-inflammatory (Song et al., 2023), anticoagulant (Sharma et al., 2022), lipid-lowering (Deng et al., 2024), and mitochondrial dysfunction-improving activities (Wang et al., 2025). As a core component of the proteoglycans abundantly present in the nervous system, CS plays a crucial role. Recent studies have shown the potential of CS in anti-AD therapy by regulating amyloid-beta (Aβ) expression through inhibition of beta-secretase 1 (BACE1), interfering with tau protein distribution and internalisation, and suppressing oxidative stress and apoptosis through multiple pathways (Gao et al., 2020; Zhao et al., 2021; Shen et al., 2024; Mycroft-West et al., 2020), making it an ideal multi-targeted therapeutic molecule. However, the inherent characteristics of CS, such as its large molecular weight and poor lipid solubility, reduce its trans-membrane transport efficiency, severely limiting its bioavailability and the full realisation of its therapeutic efficacy.

Nano-selenium (Se), a nanoparticle form of selenium, has garnered attention for its preventive and protective role in the treatment of AD (Guo et al., 2025). Studies have indicated that Se can scavenge free radicals and mitigate oxidative stress damage through its potent antioxidant capacity (Ye et al., 2023). Se can also inhibit Aβ aggregation and reduce the hyperphosphorylation of tau protein (Sun et al., 2019). These biological activities confer Se a unique application value in improving the progression of AD.

We had previously developed a novel nanomedicine with CS and Se (CS@Se) that combined the multi-target neuroprotective activity of CS with the potent antioxidant properties of Se (Ji et al., 2020). Systematic experiments in *vitro* and chemically induced AD animal models confirmed the multi-target protective effects of CS@Se. However, such chemically induced models show limited ability to recapitulate the progressive pathology and full disease spectrum of AD. Therefore, we hypothesize that CS@Se confers therapeutic benefits in APPSwe, PSEN1dE9 (APP/PS1) transgenic mice by simultaneously targeting multiple AD-associated pathologies, including Aβ deposition, neuroinflammation, tau hyperphosphorylation, and oxidative stress. To verify the aforementioned hypothesis, this study utilised APP/PS1 mice as the AD model. Our findings indicated that CS@Se significantly improves learning and memory, alleviates spatial memory deficits, reduces brain Aβ deposition, suppresses neuroinflammatory responses, and attenuates tau hyperphosphorylation in APP/PS1 mice. Subsequent molecular mechanism studies revealed that CS@Se promotes the non-amyloid hydrolysis pathway of APP by upregulating A disintegrin and metalloproteinase domain-containing protein 10 (ADAM10) expression, while synergistically exerting its multi-target neuroprotective effects by modulating glycogen synthase kinase (GSK)-3β signalling pathways. These findings suggest that **CS@Se** is a promising multi-targeted drug and lay a theoretical foundation for developing combined treatments based on CS and Se.

## 2 Materials and methods

### 2.1 Materials

CS from shark cartilage (98%, AR) was purchased from Hengjie Biopharmaceutical Co., Ltd (Jiaxing, China). CS (Mw = 3.47 kDa) was prepared in our lab. Sodium selenite (Na_2_SeO_3_, 99%, AR) and L-cysteine (L-cys, 99%, AR) were purchased from Aladdin Chemistry Co., Ltd (Shanghai, China). Histological staining kits, including kits for haematoxylin and eosin (HE) staining, Nissl staining, and glycine silver staining, as well as antibodies against APP, ADAM10, and Aβ_1-40_ were provided by Servicebio Biotechnology Co., Ltd (Wuhan, China). Choline acetyltransferase (ChAT), acetylcholinesterase (AChE), Na^+^/K^+^-ATPase assay, Glutathione peroxidase (GSH-Px), and malondialdehyde (MDA) kits were purchased from Nanjing Jiancheng Bioengineering Institute (Nanjing, China). Antibodies and glyceraldehyde-3-phosphate dehydrogenase (GAPDH) were purchased from Abcam (Cambridge, MA, United States of America), Cell Signalling Technology Inc (Danvers, MA, United States of America), and Bioss Biotechnology Co., Ltd (Beijing, China).

### 2.2 Synthesis of CS@Se

CS@Se was prepared in our laboratory as described previously (Gao et al., 2020). Briefly, CS and Na_2_SeO_3_ (CS: Na_2_SeO_3_ = 2:1, molar ratio) were dissolved in 48 mL of deionized water, followed by stirring at 30 °C for 1 h. Subsequently, 2 mL of L-cys (L-cys: Na_2_SeO_3_ = 2:1, molar ratio) was introduced to the solution and stirring continued at 30 °C for 1 h. The resulting solution was dialyzed against deionized water for 48 h using a 1,000 Da molecular weight cut-off (MWCO) membrane, with the dialysate changed every 3 h. Finally, the purified **CS@Se** solution was lyophilized, and the powder was stored at 4 °C in the dark.

### 2.3 Animals

Specific pathogen-free (SPF) male APP/PS1 mice (4 months old, Weight: 25 ± 5g, 60 in total) and SPF male C57BL/6 mice (4 months old, Weight: 25 ± 5g, 15 in total) were provided by Weishang Lituo Technology Co., Ltd (Beijing, China; Document No. SCXK-2016009) and Pengyue Experimental Animal Breeding Co., Ltd (Jinan, China; Document No. SCXK-20190,003), respectively. Mice were maintained in a laboratory (temperature: 20 °C–22 °C, humidity: 45%–55%). They had *ad libitum* access to feed and water and were randomly divided into experimental groups (15 mice per group) after 7 days of acclimation. The experimental groups and treatment plans for each group were as follows: Control group and Model group (0.9% NaCl, 10 mL/kgd), CS@Se high-dose group (H-CS@Se, 200 mg/kgd), CS@Se low-dose group (L-CS@Se, 100 mg/kgd), and Huperzine-A group (was used as a positive control, Huperzine-A, 100 μg/kgd). Oral administration was performed daily for 120 consecutive days.

### 2.4 Behavioral tests

The Morris water maze (MWM) is a common test apparatus used for assessing the learning and memory capabilities of mice (Zhu et al., 2024). The MWM experimental setup involved a cylindrical tank (110 × 30 cm, length × height) that was divided into four equal quadrants. During the experiment, the tank was filled with water up to a height of 20 cm and made opaque with titanium dioxide. The test consisted of two phases: a spatial navigation acquisition phase (days 1–5) and a probe trial phase (day 6). In the spatial navigation task, mice underwent training trials for five successive days. At the training trial, mice were released from different starting positions, and if they could not find the platform within 60 s, they were guided to the platform for 10 s to reinforce study capacity. The primary outcome measure was the latency period, which was determined as the time taken by the mice to find and climb onto the platform. After spatial navigation training, a spatial probe test was conducted. In the end, the mice were positioned in the MWM (without a platform), allowing them to swim 60 s. The number of platform crossings, time spent in the target quadrant, and swimming trajectory were recorded and analyzed by the MWM system.

The Y-maze task was used to evaluate the discriminative studying and working memory of the mice (Lee et al., 2025). The apparatus consisted of three closed arms (30 × 8 × 15 cm) arranged at 120-degree angles, with a central area forming an equilateral triangle (side length: 8 cm). The arms were labelled No. 1, No. 2, and No. 3. The number of times mice entered each channel within 10 min was recorded. An alternation was defined as three consecutive entries into three different arms (e.g., 1-2-3, 2-1-3, or 3-1–2). For each mouse, the total number of arm entries (*N*) and complete alternations (n) were used to calculate the percentage alternation (*P*) with the following formula: $P=\frac{n}{N}\times 100\%$ .

The new object recognition (NOR) experiment was adopted to evaluate the learning and visual-recognition capabilities of the mice (Lourenco et al., 2019) (Liu et al., 2022). The experiment was conducted over 3 days in an activity box (50 × 50 × 50 cm). On Day 1, the mice were allowed to freely explore the activity box for 5 min. On Day 2, two identical cylinders (A_1_ and A_2_) were placed in the activity box. The mice were given 5 min to explore and familiarise themselves with the objects. The time taken to explore the two identical cylinders (t_A1_ and t_A2_) was recorded. On Day 3, object A_2_ was replaced with a new object (B, a cube), and the mice were given 5 min to explore the objects. The time taken to explore the new object B (t_B_) and A_1_ (t_A1_) was recorded. The preference index (PI) was calculated as follows: PI = t_B_/(t_A1_ + t_B_).

The nest-building test (NBT) was designed to measure the nesting behaviour and learning capacity of the mice (Shi et al., 2018). Mice were singly housed in standard cages (25 × 15 × 14 cm), with six square sheets of soft paper (4 × 4 cm) evenly distributed on the bedding as nesting material. The experiment was conducted overnight from 20:00 to 08:00 the following day, lasting for 12 h, during which normal feed and water were provided to the mice. After the experiment, the nesting structures were photographed and scored for quality using a modified four-point scale: 0 = no nest; 1 = flat nest (paper torn but not formed); 2 = simple nest (paper shredded and loosely gathered); 3 = intermediate nest (shredded paper formed into a cup shape); 4 = complete nest (shredded paper formed into a well-defined, high-walled cup shape) (Shan et al., 2023). All scoring procedures were performed blindly to eliminate bias.

All behavioral sessions were recorded using a digital video camera mounted above the apparatus. The recorded videos were analyzed using TopScan software (version 3.0; Clever Sys Inc., Reston, VA, United States of America).

### 2.5 Brain tissue preparation

Upon conclusion of the behavioural experiments, the mice were administered sodium pentobarbital (50 mg/kg). Subsequently, approximately 50 mL of pre-chilled 0.9% NaCl was used to eliminate the blood within the body. Rapid perfusion was conducted using pre-chilled 4% paraformaldehyde. The entire brain was isolated and fixed at 4 °C for 24 h. Subsequently, paraffin embedding of the brain was performed. For histological analysis, coronal sections were cut at Coronal sections were cut at 5 or 20 μm thickness using a microtome (Leica RM2255) and collected in a 1:6 series. For hippocampal analysis, sections spanning bregma levels from −1.70 mm to −2.54 mm (containing the dorsal hippocampus) were selected. For cortical analysis, sections spanning bregma levels from +1.0 mm to −1.0 mm were selected. Anatomical locations were confirmed according to the mouse brain atlas of Paxinos and Franklin.

### 2.6 Transmission electron microscopy

After the behavioural assessments, mice were administered sodium pentobarbital for anaesthesia, which was followed by perfusion with 0.1 mol/L pre-chilled phosphate-buffered saline. After perfusion, the hippocampi were carefully isolated and fixed overnight in glutaraldehyde solution (2.5%). Then, the collected tissue was fixed in an osmium tetroxide system (1%) for 2 h. After dewatering through a graded acetone series, epitaxial sections of the tissue mass were obtained using an ultramicrotome. Finally, the ultrastructure of the hippocampal neurons was investigated by using a JEM-1200 EX II TEM from Japan Electron Optics Laboratory (Jeol, Japan).

### 2.7 Measurement of biochemical factors

After behavioural testing, the hippocampi of mice were carefully isolated and stored at −80 °C. The tissues to be tested, such as the hippocampus, were homogenised according to the kit instructions. The homogenate was centrifuged at 3,000 rpm (15 min), and the supernatant was collected for subsequent analysis. The supernatant was diluted ten-fold with physiological saline for further analysis. The activities and levels of ChAT, AChE, Na^+^/K^+^-ATPase, GSH-Px, as well as MDA were assessed by test kits in accordance with the manufacturers’ protocols. All biochemical data were normalized to total protein content. Total protein concentration in each sample was measured using the BCA Protein Assay Kit (Beyotime, Cat# P0012) with bovine serum albumin (BSA) as the standard. Absorbance was read at 562 nm using a microplate reader (BioTek, ELx808). The results of all biochemical assays were expressed as per milligram of total protein (e.g., U/mg protein for enzyme activities).

### 2.8 Morphological detection and analysis

The brain samples were sectioned into 5- or 20-μm-thick slices. Coronal hippocampal slices were mounted onto glass slides. The entire brain tissue sections were used for staining procedures such as Nissl staining and silver impregnation staining using glycine (Palmgren) as well as immunohistochemistry (IHC) and immunofluorescence (IF) analyses. Image analysis and quantification were performed using ImageJ 1.8.0 software (Bio-Rad Laboratory, California, United States of America).

### 2.9 Western blot

Hippocampal and cortical tissues were collected and homogenised in pre-chilled radioimmunoprecipitation assay (RIPA) buffer. The supernatant was collected by centrifugation at high speed (15 min, 12,000 rpm, 4 °C). Protein concentration was determined by BCA assay method. Protein was further denatured by mixing it with RIPA buffer and 5× loading buffer (100 °C, 5 min). The denatured proteins (20–30 μg per lane) were separated by 12% sodium dodecyl sulfate-polyacrylamide gel electrophoresis (SDS-PAGE) at a constant voltage of 80 V through the stacking gel and 120 V through the resolving gel. Subsequently, the separated proteins were electrotransferred onto polyvinylidene fluoride (PVDF) membranes using a wet transfer system at 300 mA constant current for 90 min at 4 °C. Subsequently, protein bands were blocking (5% skimmed milk, 1 h at 20 °C–25 °C), application of primary antibodies (including: anti-APP, 1:1,000; anti-ADAM10, 1:600; anti-BACE-1, 1:1,000; anti-C99/83, 1:4,000; anti-Aβ, 1:2000; anti-1β, 1:2000; anti-IL-6, 1:2000; anti-TNF-α, 1:5,000; anti-GSK-3β, 1:10,000; anti-P-GSK-3β, 1:1,000; anti-PP2A, 1:10,000; anti-Tau, 1:5,000; anti-p-Tau(Ser396), 1:10,000; anti-p-Tau(Ser404), 1:1,000; anti-p-Tau(Ser199), 1:5,000; anti-p-Tau(Thr205), 1:1,000 and anti-β-actin or anti-GAPDH, 1:5,000 as an internal loading control) overnight at 4 °C and secondary antibodies (1:5,000) for 1 h at room temperature (20 °C–25 °C), and development. Quantitative evaluation was performed using ImageJ 1.8.0 software (Bio-Rad Laboratory, California, United States of America). The relative expression level of each target protein was normalized to the corresponding internal control (β-actin or GAPDH) and expressed as fold-change relative to the control group.

### 2.10 Statistical analysis

Statistical analyses were performed using GraphPad Prism9.0 (GraphPad Software Inc., California, United States of America). One-way ANOVA and *t*-tests were performed to compare group differences. Data were presented as mean ± standard deviation (SD). *P* < 0.05 was considered statistically significant between groups.

## 3 Results

### 3.1 CS@Se improves learning and memory deficits in APP/PS1 mice

In behavioural testing experiments, we first evaluated the efficacy of CS@Se in mitigating learning and memory deficits. As shown in Figures 1B–E and Supplementary Figure S2, in the MWM test, CS@Se treatment significantly shortened the escape latency, increased platform crossings, and prolonged the target quadrant time in comparison with the corresponding values in the Model group, and all improvements exhibited a dose-dependent relationship. The NOR and Y-maze tests leverage the innate exploratory behaviour of mice to evaluate recognition memory. After CS@Se treatment, the mice showed significant increments in both the PI for novel objects (Figure 1F) and the spontaneous alternation ratio score (Figure 1G). These results indicate that CS@Se could effectively enhance learning abilities and mitigate cognitive dysfunction in AD mice. To evaluate the effects of CS@Se on motor function in APP/PS1 mice, we conducted the NBT, another behavioural assessment. The NBT can reflect social interactions and daily activity capabilities in mice. As shown in Figures 1H,I, CS@Se-treated mice constructed more complete nests and achieved higher scores. These results indicate that CS@Se effectively enhances spontaneous activity in APP/PS1 mice. In conclusion, these behavioural test results demonstrated that CS@Se could significantly improve learning, memory, and cognition in APP/PS1 mice, and increase their spontaneous activity.

**FIGURE 1:**
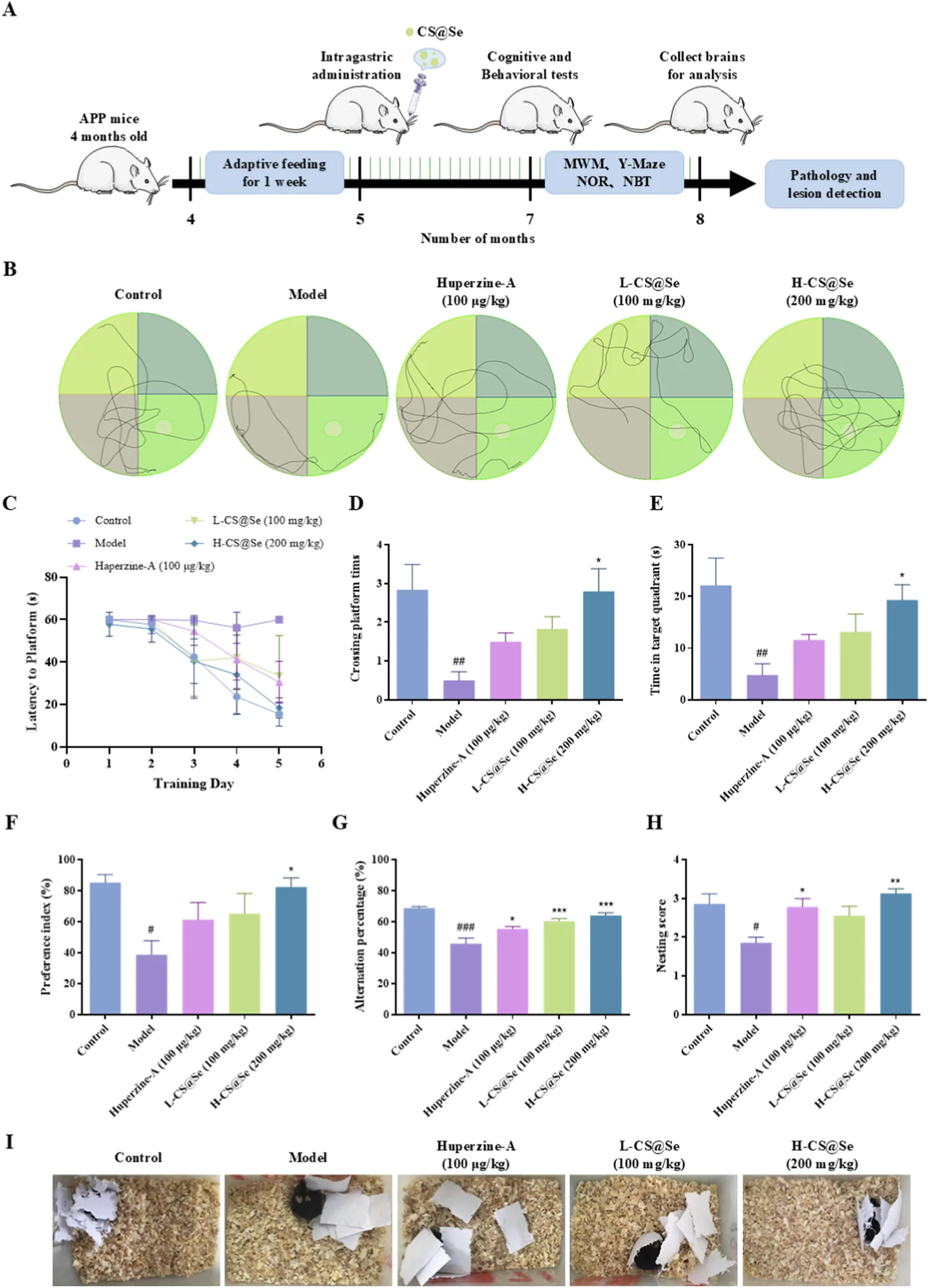
Effects of CS@Se on behavioural cognitive impairment in APP/PS1 transgenic mice. **(A)** Protocols and treatment plans for the animal experiments. **(B)** Representative diagrams of Morris water maze (MWM) trajectories for each group. **(C)** Escape latency of each group during the first 5 days of the MWM experiment. **(D,E)** Number of crossings of the target platform quadrant and time spent in the platform quadrant on day 6 of the MWM experiment. **(F)** Preference index calculated in the new object recognition (NOR) experiment. **(G)** Spontaneous alternation ratio of mice in the Y-maze test. **(H,I)** Representative nest-building test (NBT) and nest scores for each group of mice. Each experiment was repeated three times. The data are reported as the mean ± standard deviation (n = 6): ^#^ *p* < 0.05, ^##^ *p* < 0.01 vs. Control group; ^*^ *p* < 0.05, ^**^ *p* < 0.01, ^***^ *p* < 0.001 vs. Model group, significantly different from the Control group.

### 3.2 CS@Se modulates cholinergic function, attenuates oxidative stress damage, and improves energy metabolism in APP/PS1 mice

Dysfunction of the cholinergic system is one of the key pathological features of AD (Zhao et al., 2024; Wu et al., 2024). Therefore, we measured the activities of AChE and ChAT in brain tissue from APP/PS1 mice to assess the effect of CS@Se. As shown in Figures 2A,B, the Model group exhibited significantly elevated AChE activity and markedly reduced ChAT activity in comparison with the Control group, indicating dysfunction of cholinergic neurotransmission that was consistent with AD pathology. After the CS@Se intervention, AChE activity was significantly reduced and ChAT activity was effectively restored, indicating that CS@Se can improve cholinergic dysfunction in APP/PS1 mice. Oxidative stress is another critical factor in AD pathogenesis (Tan et al., 2024; Chen et al., 2024). We evaluated the effect of CS@Se by measuring the content and activity of MDA and GSH-Px, respectively. As shown in Figures 2C,D, the MDA levels were significantly higher and the GSH-Px activity was markedly lower in the Model group, indicating marked oxidative damage. After treatment with CS@Se, MDA levels significantly reduced and GSH-Px activity was restored. The Na^+^/K^+^-ATPase plays a central role in maintaining neuronal membrane potential and intracellular homeostasis, and decreased activity of this enzyme is closely associated with neuronal metabolic dysfunction (Petrushanko et al., 2022). Our results showed that the activity of Na^+^/K^+^-ATPase was significantly reduced in the brain tissue of APP/PS1 mice, while all CS@Se treatment groups showed significantly increased activity of this enzyme. In summary, this study demonstrated that CS@Se effectively reduces AChE activity and MDA content while enhancing the activity of ChAT, GSH-Px, and Na^+^/K^+^-ATPase. Thus, CS@Se could inhibit lipid peroxidation, improve cholinergic transmission, and enhance neuronal metabolic function.

**FIGURE 2:**
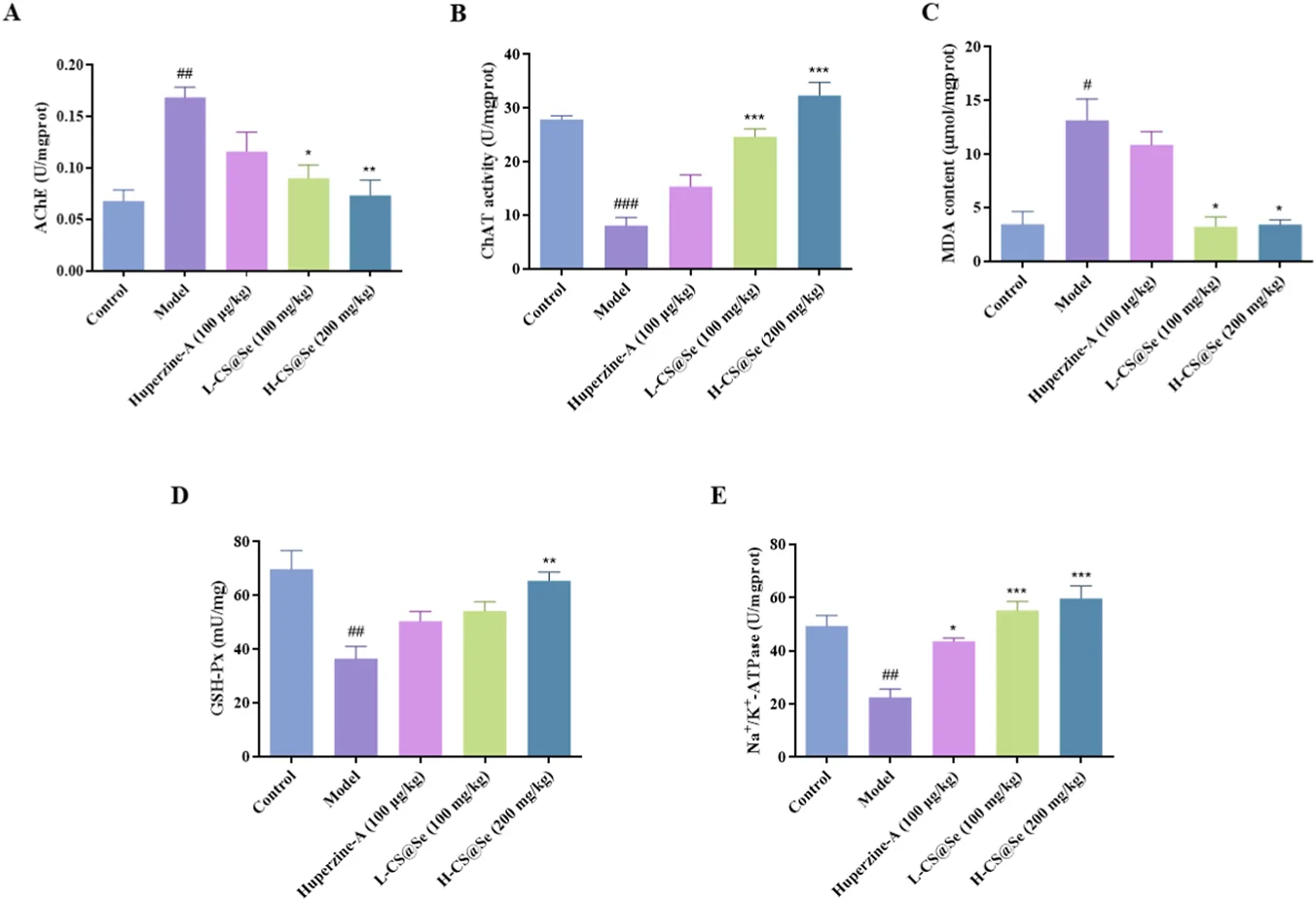
Effects of CS@Se on cholinergic function, oxidative stress damage, and energy metabolism in APP/PS1 transgenic mice. **(A-E)** Effects of CS@Se on the levels of acetylcholinesterase (AChE), choline acetyltransferase (ChAT), malondialdehyde (MDA), glutathione peroxidase (GSH-Px), and Na^+^/K^+^-ATPase. Each experiment was repeated three times. The data are reported as the mean ± standard deviation (n = 6): ^#^ *p* < 0.05, ^##^ *p* < 0.01, ^###^ *p* < 0.001 vs. Control group; ^*^ *p* < 0.05, ^**^ *p* < 0.01, ^***^ *p* < 0.001 vs. Model group, significantly different from the Control group.

### 3.3 CS@Se protects the morphology, function, and ultrastructure of synapses

To assess the neuroprotective effects of CS@Se, we performed HE staining on the hippocampus and evaluated the morphological changes. As shown in Figure 3A, neurons in the CA1, CA3, and dentate gyrus (DG) regions of APP/PS1 mice exhibited significantly reduced cell width and loosely arranged structures. Some neurons demonstrated nuclear condensation (red arrows) and cellular oedema (green arrows), indicating the occurrence of damaging neuronal lesions. After CS@Se treatment, neuronal cell arrangement in these regions was restored to orderly patterns, and cell morphology improved markedly, with a significant reduction in nuclear condensation and cellular oedema. These findings suggest that CS@Se could reverse neuronal damage. Nissl bodies are a key indicator for evaluating neuronal function (Zhang et al., 2025; Liu et al., 2025). The protective effect of CS@Se on neuronal function was assessed by Nissl staining. Control mice exhibited abundant Nissl bodies (Supplementary Figure S3) in the CA1, CA3, and DG regions of neurons (purple arrows), whereas APP/PS1 mice showed loss of Nissl bodies in these neuronal regions (yellow arrows). However, after treatment with CS@Se, the number of Nissl bodies recovered (purple arrows), indicating that CS@Se had a restorative effect on neuronal function. Furthermore, the ultrastructure, synaptic density, and mitochondrial architecture of the hippocampal neurons of mice were examined using transmission electron microscopy. As shown in Figures 3B–E, mice in the Model group exhibited myelin damage (red arrows), thinning of the postsynaptic dense band (yellow arrows), mitochondrial membrane rupture, and ridge degeneration (purple arrows). After CS@Se treatment, myelin sheaths were compactly aligned, postsynaptic densities became thicker, and mitochondrial damage was significantly reduced. Collectively, these results indicate that CS@Se shows neuroprotective effects in AD mice by preserving hippocampal neuron morphology and function and thereby promoting neuronal survival.

**FIGURE 3:**
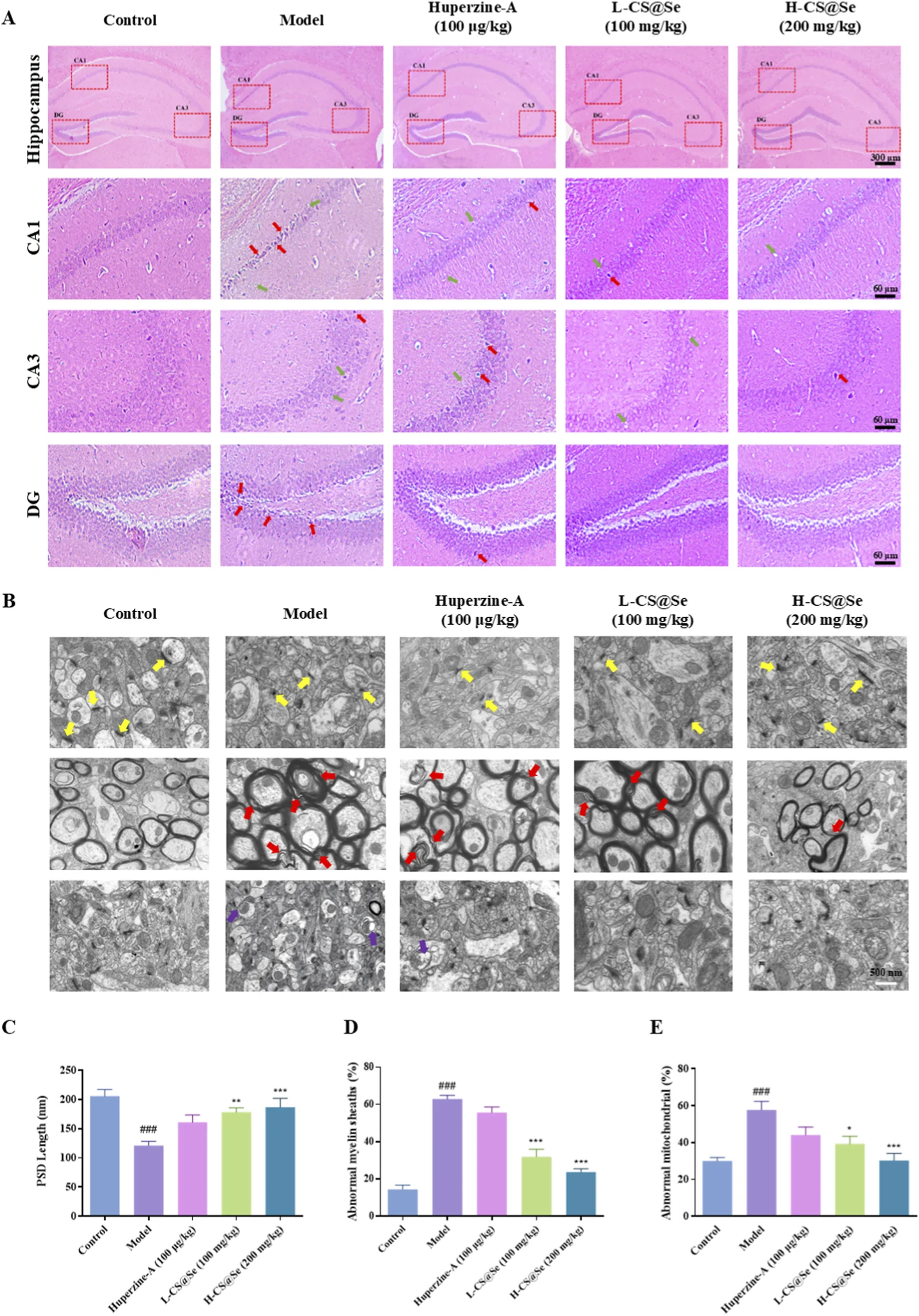
Effects of CS@Se on hippocampal neurons of APP/PS1 transgenic mice. **(A)** Haematoxylin and eosin (HE) staining. Nuclear condensation is indicated with red arrows and cellular oedema is indicated with green arrows. The scale bar was 300 or 60 μm. **(B)** Transmission electron microscopy images of myelin sheaths, synapses, and mitochondria in the hippocampus of APP/PS1 mice. Myelin damage is indicated by red arrows. The postsynaptic dense bands are indicated by yellow arrows. Mitochondrial membrane rupture and ridge degeneration are indicated by purple arrows. **(C-E)** Indicator statistics chart. Each experiment was repeated three times. The data are reported as the mean ± standard deviation (n = 3): ^###^ *p* < 0.001 vs. Control group; ^*^ *p* < 0.05, ^**^ *p* < 0.01, ^***^ *p* < 0.001 vs. Model group, significantly different from the Control group.

### 3.4 CS@Se improves aβ deposition and expression of APP metabolism-related factors in the hippocampal and cortical regions of APP/PS1 mice

To evaluate the activity of CS@Se in Aβ degradation *in vivo*, we performed immunohistochemistry (IHC) staining and Western blot analyses. Mice in the Model group exhibited severe Aβ plaque deposition in the hippocampal and cortical regions in comparison with the Control group (Figure 4A). After CS@Se treatment, the number and density of Aβ plaques in these brain regions significantly reduced in a dose-dependent manner. To further investigate the molecular mechanisms by which CS@Se reduces Aβ deposition in APP/PS1 mice, we measured the expression levels of Aβ metabolism-related proteins in the hippocampal and cortical regions by performing Western blotting. The results (Figures 4E–H) show that CS@Se could effectively downregulate the expression of APP, BACE-1, and C99/83 in hippocampal and cortical tissues while simultaneously upregulating the expression of ADAM10. These results indicate that CS@Se could reduce Aβ deposition in the hippocampal and cortical regions of AD mice by modulating the expression of related proteins in APP metabolic pathway and inhibiting Aβ generation.

**FIGURE 4:**
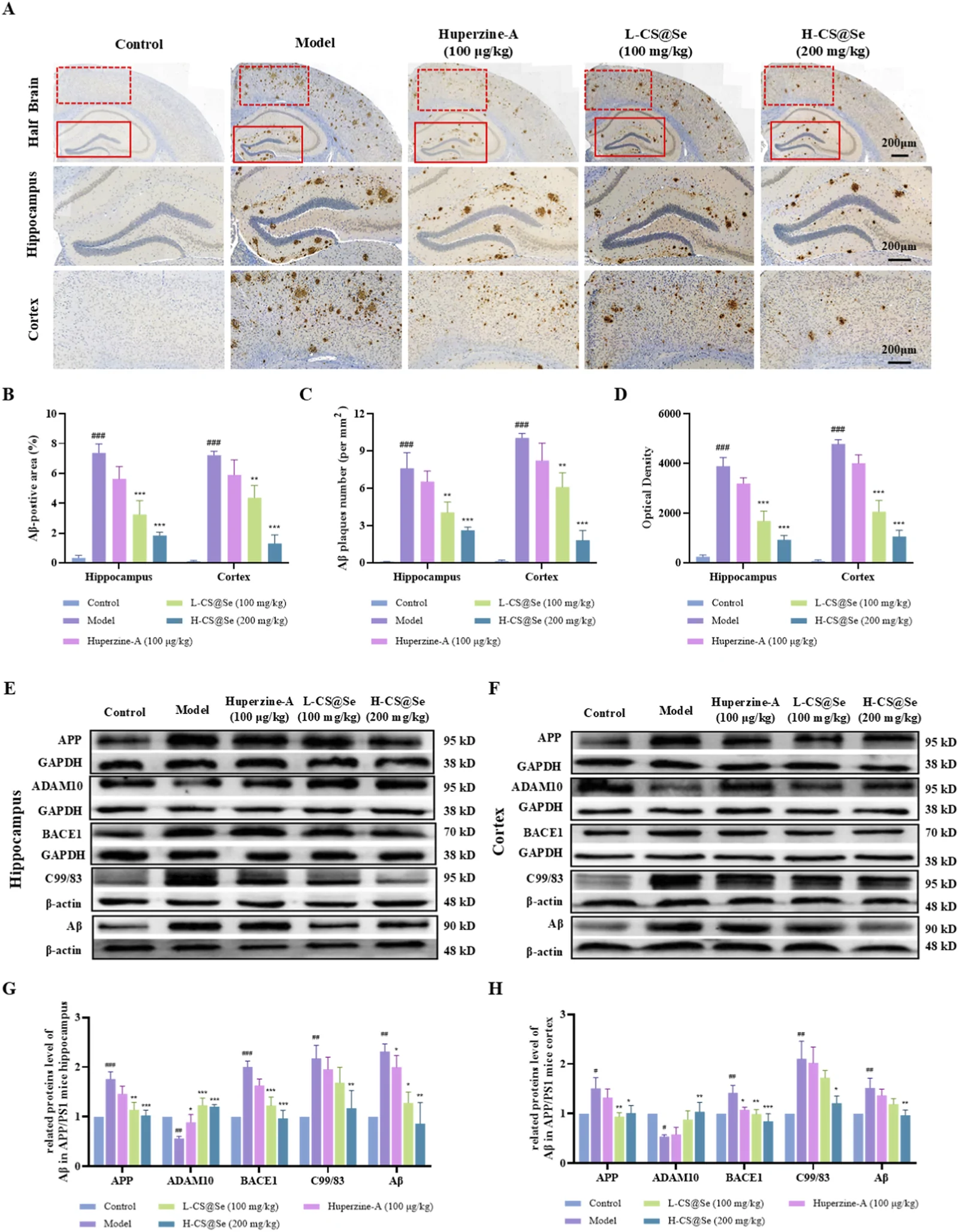
Effects of CS@Se on Aβ deposition and APP metabolic-related factors in the hippocampus and cortical regions of APP/PS1 mice. **(A)** Immunohistochemical (IHC) staining images of Aβ in the hippocampus and cortex. The scale bar was 200 μm. **(B-D)** Quantitative analysis of Aβ IHC staining. Effects of CS@Se on the expressions of APP-related metabolic factors, including ADAM10, BACE1, C99/83, and Aβ in the hippocampus **(E)** and cortical regions **(F)** of APP/PS1 mice. The statistical histograms for these effects are shown in **(G,H)**. Each experiment was repeated three times. The data are reported as the mean ± standard deviation (n = 3): ^#^ *p* < 0.05, ^##^ *p* < 0.01, ^###^ *p* < 0.001 vs. Control group; ^*^ *p* < 0.05, ^**^ *p* < 0.01, ^***^ *p* < 0.001 vs. Model group, significantly different from the Control group.

### 3.5 CS@Se could effectively suppress the aberrant activation of glial cells and mitigate inflammatory responses in the hippocampal and cortical regions of APP/PS1 mice

Microglia and astrocytes are the principal immune regulatory cells in the central nervous system (Keren-Shaul et al., 2017). Under pathological stimuli such as Aβ, these cells release inflammatory mediators, thereby triggering neuroinflammation (Habib et al., 2020; Leng and Edison, 2021). In this study, we performed IF staining to examine the effects of CS@Se on Aβ deposition and the activation of microglia (ionised calcium binding adaptor molecule 1, Iba-1) and astrocytes (glial fibrillary acidic protein, GFAP) in the hippocampal and cortical regions APP/PS1 mice. As shown in Figures 5A–D, mice in the Model group showed significantly elevated levels of Iba-1 and GFAP in the hippocampal and cortical regions, indicating abnormal activation of glial cells in these areas and suggesting neuroinflammation. Notably, the intensity of Iba-1 and GFAP positivity was significantly higher in the areas surrounding the Aβ deposits than in non-deposited regions, indicating that Aβ aggregation directly drives glial cell activation. After CS@Se treatment, the levels of Iba-1 and GFAP, as well as Aβ deposition, were significantly lower in the hippocampal and cortical regions of mice. Furthermore, extensive research has shown that pathological proteins such as Aβ can activate glial cells, causing uncontrolled neuroinflammation and thereby exacerbating neuronal damage (Du et al., 2019; Zhao et al., 2023). To determine whether CS@Se could modulate neuroinflammatory responses, we assessed the expression of proinflammatory factors of the hippocampal and cortical regions APP/PS1 mice by Western blot analysis. After treatment with CS@Se, the expression of inflammatory proteins was significantly reduced (Figures 5E–H). Thus, CS@Se could suppress Aβ deposition and abnormal activation of glial cells in the hippocampal and cortical regions, thereby alleviating neuroinflammation and exerting a protective effect.

**FIGURE 5:**
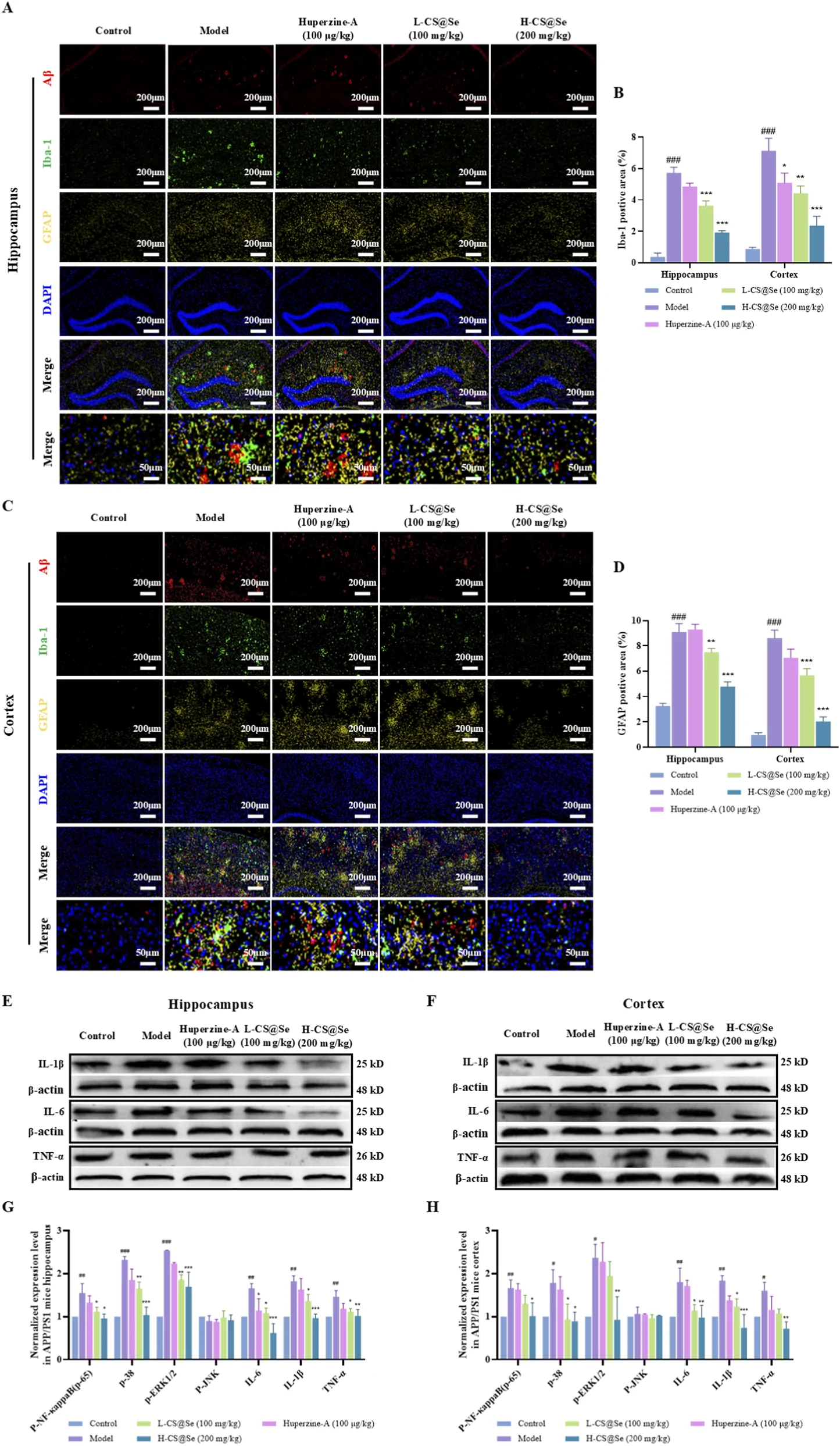
Effects of CS@Se on neuroinflammation **(A)** and **(C)** Effects of CS@Se on the expression of microglia (ionised calcium binding adaptor molecule 1, Iba-1) and astrocytes (glial fibrillary acidic protein, GFAP) and Aβ deposition in the hippocampal and cortical regions of APP/PS1 mice. The statistical histograms for these effects are shown in **(B)** and **(D) .(E,F)** Effects of CS@Se on the expression of inflammation-related factors in the hippocampus and cortical regions of APP/PS1 mice. The statistical histograms for these effects are shown in **(G,H)**. Each experiment was repeated three times. The data are reported as the mean ± standard deviation (n = 3): ^#^ *p* < 0.05, ^##^ *p* < 0.01, ^###^ *p* < 0.001 vs. Control group; ^*^ *p* < 0.05, ^**^ *p* < 0.01, ^***^ *p* < 0.001 vs. Model group, significantly different from the Control group.

### 3.6 CS@Se effectively suppresses excessive phosphorylation of tau protein and reduces neurofibrillary tangles in the hippocampal and cortical regions of APP/PS1 mice

Neurofibrillary tangles (NFTs) are a hallmark neuropathological feature of AD (DeTure and Dickson, 2019; Ising et al., 2019). To evaluate the effect of CS@Se on NFT formation, we examined the hippocampal and cortical regions of APP/PS1 transgenic mice by Gallyas silver staining (GALLYAS). In comparison with the Control group, mice in the Model group exhibited significantly increased NFT density (Figure 6A) in the hippocampal CA1, CA3, and DG regions (indicated by red arrows). After treatment with CS@Se, the number of NFTs in these regions reduced markedly. The results of GALLYAS for cortical regions (Figure 6B) further revealed a significant reduction in NFT deposition within the reticular activating system, associative cortex area 1 (AS1), and basolateral amygdala (BLA) regions of the CS@Se-treated mice. These results indicate that CS@Se could readily alleviate neurodegeneration associated with tau pathology.

**FIGURE 6:**
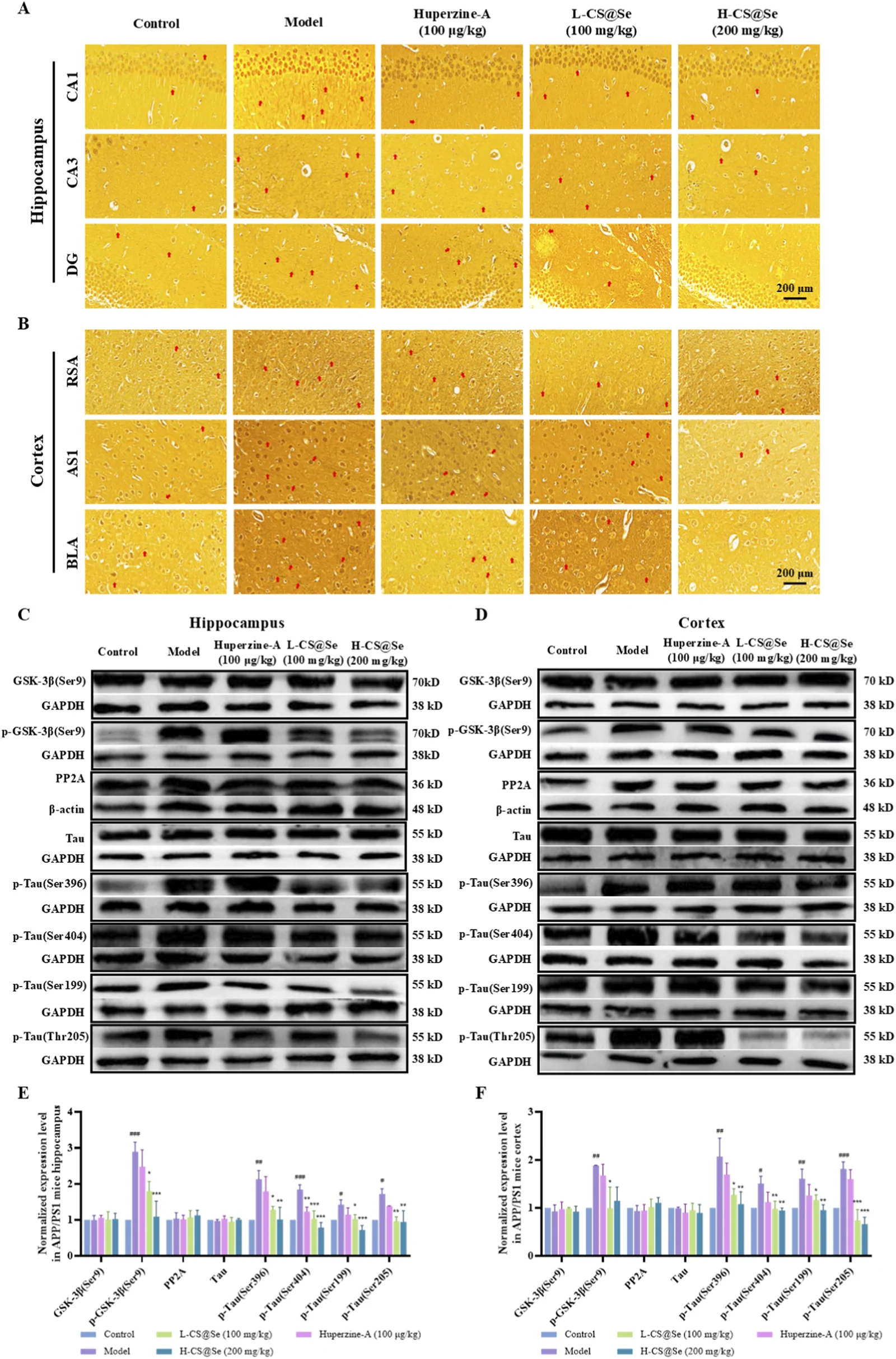
Effects of CS@Se on tau protein hyperphosphorylation. **(A,B)** Gallyas silver staining (GALLYAS) in hippocampal and cortical regions; neurofibrillary tangles (NFTs) are indicated with red arrows. The scale bar was 200 μm. **(C,D)** Effects of CS@Se on the expression of GSK-3β-Tau pathway-related proteins in the hippocampus and cortical regions of APP/PS1 mice. The statistical histograms for these effects are shown in **(E,F)**. Each experiment was repeated three times. The data are reported as the mean ± standard deviation (n = 3): ^#^ *p* < 0.05, ^##^ *p* < 0.01, ^###^ *p* < 0.001 vs. Control group; ^*^ *p* < 0.05, ^**^ *p* < 0.01, ^***^ *p* < 0.001 vs. Model group, significantly different from the Control group.

NFTs are intimately associated with abnormal hyperphosphorylation and aggregation of tau protein (Hardy and Selkoe, 2002). Experimental evidence indicates that tau protein undergoes specific hyperphosphorylation at the S396, S404, T205, and S199 sites, a process primarily driven by GSK-3β (Goedert et al., 1989). Notably, inhibition of p-GSK-3β expression has been shown to significantly reduce phosphorylation levels at these sites, suggesting that the GSK-3β-Tau phosphorylation axis plays a critical role in NFTs formation (Wang et al., 2022; Scarpa et al., 2021). We also analysed the expression of relevant proteins by Western blot analyses. The model mice exhibited significantly elevated phosphorylation levels of tau protein at sites S396, S404, T205, and S199 in the hippocampal and cortical regions in comparison with the Control group (Figures 6C–F). After treatment with CS@Se, phosphorylation levels at all sites in these regions reduced significantly. In addition, p-GSK-3β (Ser9) levels were significantly elevated in both the hippocampus and cortex of the Model group, while protein phosphatase 2 A (PP2A) expression remained unchanged. Thus, abnormal activation of GSK-3β is a key factor driving excessive tau phosphorylation. After CS@Se treatment, p-GSK-3β levels were significantly reduced in both the hippocampus and cortical regions. In summary, our findings showed that CS@Se could inhibit GSK-3β activity in the hippocampal and cortical regions of APP/PS1 mice, restore the kinase-phosphatase balance, and thereby alleviate excessive phosphorylation of tau protein.

## 4 Discussion

Alzheimer’s disease (AD) is a multifactorial neurodegenerative disorder characterized by progressive cognitive decline and a complex network of pathological alterations, including Aβ accumulation, tau hyperphosphorylation, oxidative stress, neuro-inflammation, and synaptic dysfunction (Selkoe and Hardy, 2016; Long and Holtzman, 2019). Although recent advances in anti-amyloid monoclonal antibody therapies have provided evidence for disease-modifying approaches, their overall clinical benefits remain limited, underscoring the need for complementary therapeutic strategies capable of simultaneously targeting multiple pathological processes (Farrell et al., 2026). In this study, we investigated the therapeutic efficacy of CS@Se in APP/PS1 transgenic mice and demonstrated that CS@Se effectively alleviated cognitive deficits and ameliorated multiple AD-related pathological abnormalities, supporting its potential as a multi-target therapeutic candidate for AD.

Cognitive decline in AD is closely associated with synaptic loss and neuronal dysfunction (Wei et al., 2025). Synaptic dysfunction represents an early and critical event in AD progression (Selkoe, 2002). Therefore, protection of synaptic architecture may contribute substantially to the improvement of cognitive performance. Our results showed that CS@Se could significantly improve behavioral performance, restore neuronal morphology, and preserve hippocampal synaptic ultrastructure in APP/PS1 mice. These findings suggest that CS@Se exerts neuroprotective effects beyond reducing pathological markers, potentially by maintaining neuronal structural integrity and synaptic function. Oxidative stress is a key contributor to AD pathogenesis, promoting mitochondrial dysfunction, neuronal damage, and inflammatory activation (Bai et al., 2022). Our previous studies have shown that CS@Se reduces oxidative stress markers in D-gal/AlCl_3_-induced AD mice (Ji et al., 2020). In the present study, CS@Se could enhance GSH-Px activity, reduce MDA accumulation, and restore Na^+^/K^+^-ATPase activity, indicating improved antioxidant capacity and neuronal energy metabolism in the APP/PS1 mice model. Furthermore, the recovery of ChAT activity and suppression of AChE activity suggest that CS@Se could alleviate cholinergic dysfunction, which is closely associated with cognitive impairment in AD. These results indicate that the antioxidant and metabolic regulatory functions of CS@Se may provide a favorable environment for neuronal survival.

Aβ accumulation, neuroinflammation and tau hyperphosphorylation are major pathological hallmarks of AD (La Barbera et al., 2025). Unlike therapeutic strategies targeting only a single pathological component, CS@Se could regulate APP metabolic pathway-related proteins and inhibit Aβ deposition in hippocampal and cortical regions. This effect may decrease Aβ-associated neurotoxicity by limiting amyloid accumulation and disrupting the pathological interaction between Aβ, oxidative stress, and inflammation. Neuroinflammation is another important driver of AD progression. Excessive activation of astrocytes and microglia promotes the release of pro-inflammatory cytokines and further aggravates neuronal injury. In this study, CS@Se suppressed abnormal glial activation and reduced inflammatory responses, indicating that CS@Se may interrupt the vicious cycle between inflammation and neurodegeneration. Moreover, CS@Se could inhibit abnormal phosphorylation of tau protein through the GSK-3β-Tau phosphorylation pathway, thereby suppressing the formation of neuronal fibre tangles. These results indicate that the simultaneous modulation of amyloid, neuroinflammatory, and tau pathways by CS@Se may provide a comprehensive neuroprotective milieu that counteracts the multifaceted pathology of AD.

It should be noted that our study still has some limitations. On the one hand, the long-term safety and pharmacokinetics of CS@Se were not examined, and further research is needed before clinical translation can be achieved. On the other hand, the blood-brain barrier permeability of CS@Se was not directly evaluated. Future studies should address these limitations through pharmacokinetic analysis, chronic toxicity assessments, and investigations into blood-brain barrier permeability. In summary, this study provides *in vivo* evidence that CS@Se represents a promising multi-target nanotherapeutic strategy for AD treatment. The combination of selenium-mediated antioxidant activity and chondroitin sulfate-based biological functions enables CS@Se to simultaneously modulate several key pathological processes involved in AD progression. These findings expand our understanding of nanomaterial-based therapeutic approaches and support further investigation of CS@Se as a potential disease-modifying intervention for AD.

## Data Availability

The original contributions presented in the study are included in the article/Supplementary Material, further inquiries can be directed to the corresponding authors.
