# Supplementary material for: A coordinated multi-target approach based on chondroitin sulphate nano-selenium mitigates Alzheimer’s disease pathologies in APP/PS1 mice
Source: Front Pharmacol. 2026 Sep 15;17:1936198. doi: 10.3389/fphar.2026.1936198 (PMC13619492; doi:10.3389/fphar.2026.1936198)
Supplement: Supplementary file 1 [file Supplementaryfile1.docx]

**Supplementary Material for**

**A Coordinated Multi-Target Approach Based on Chondroitin Sulphate Nano-selenium Mitigates Alzheimer's Disease Pathologies in APP/PS1 Mice**

Dongsheng Ji ^a1^, Jing Zhao ^b1^, Xinhui Huang ^a,c1^, Xuan Liu ^d1^, Qirong Yang ^c^, Xinfeng Li^c^, Lei Yan ^a,*^, Debo Gao ^a,*^, Yuliang Xiao^a,*^

^a^ Shandong Cancer Hospital and Institute, Shandong First Medical University & Shandong Academy of Medical Sciences, Jinan, 250117, Shandong, China

^b^ Department of Pharmacy, Shandong Provincial Taishan Hospital, Taian 271000, Shandong, China

^c^ School of Pharmaceutical Sciences, Shandong First Medical University & Shandong Academy of Medical Sciences, Jinan, 250117, Shandong, China

^d^ Department of Hematology, Qilu Hospital, Cheeloo College of Medicine, Shandong University, Jinan, Shandong 250012, China

^1^ Authors equally contributed to this work.

^*^ Corresponding author:

E-mail addresses: yanlei sd@163.com (L.Yan), gdb1996cr7@163.com (D. Gao), xiaoyl@sdfmu.edu.cn (Y. Xiao)

**Table of Contents**

| **Supporting** **Graphs** |  |
| --- | --- |
| **Figure S1.** Characterization diagram of **CS@Se** | S3 |
| **Figure S2.** Effects of **CS@Se** on behavioral cognitive impairment in APP/PS1 transgenic mice | S3 |
| **Figure S3.** Nissl staining | S4 |
| **Table S1** Reagents/Test Kits | S5 |
|  |  |

Fig. S1 Characterization diagram of **CS@Se**. (A) Weight-average molecular weight of CS. (B) FT-IR spectra of CS and **CS@Se**. (C) TEM image of **CS@Se** nanoparticles. (D) H^1^-NMR spectra of CS and **CS@Se**.

Fig. S2 Effects of **CS@Se** on behavioral cognitive impairment in APP/PS1 transgenic mice. Time spent in the target quadrant by each group during the 6th day of the MWM experiment. All experiments were repeated three times. Data are expressed as the mean ± SD: ^###^*p* < 0.001 vs Control group; ^*^*p* < 0.05, ^**^*p* < 0.01 vs Model group, significantly different from the control.

Fig. S3 Nissl staining was performed with Nissl bodies indicated by purple arrows, loss of Nissl bodies indicated by yellow arrows. The scale bar was 300 μm or 60 μm.

Table S1. Reagents/Test Kits

| Reagents/Test Kits | Vendor | Catalog # |
| --- | --- | --- |
| ChAT Assay Kit | Nanjing Jiancheng | A079-1-1 |
| AChE Assay Kit | Nanjing Jiancheng | A024-1-1 |
| Na⁺/K⁺-ATPase Assay Kit | Nanjing Jiancheng | A070-2-1 |
| GSH-Px Assay Kit | Nanjing Jiancheng | A005-1-1 |
| GSH-Px Assay Kit | Nanjing Jiancheng | A005-1-1 |
| MDA Assay Kit | Nanjing Jiancheng | A003-1-1 |
| anti-APP | Abcam | ab126732 |
| anti-ADAM10 | Abcam | ab124695 |
| anti-BACE-1 | Abcam | ab183612 |
| anti-C99/83 | Abcam | ab32136 |
| anti-Aβ | Abcam | ab2539 |
| anti-IL-1β | Abcam | ab9722 |
| anti-IL-6 | Abcam | ab214429 |
| anti-TNF-α | Abcam | ab6671 |
| anti-GSK-3β | Abcam | ab93926 |
| anti-P-GSK-3β | Abcam | ab131097 |
| anti-PP2A | Abcam | ab32141 |
| anti-Tau | Abcam | ab76128 |
| anti-p-Tau(Ser396) | Abcam | ab32057 |
| anti-p-Tau(Ser404) | Abcam | ab92676 |
| anti-GAPDH | Cell Signaling Technology | 5174 |
| anti-β-actin | Cell Signaling Technology | 3700 |
| HRP-conjugated Goat Anti-Rabbit IgG (H+L) | Bioss Biotechnology | bs-40295G-HRP |
| BCA Protein Assay Kit | Beyotime | P0012 |
| RIPA Lysis Buffer (Strong) | Beyotime | P0013B |
| L-Cysteine | Aladdin Chemistry | 52-90-4 |
| Sodium Selenite | Aladdin Chemistry | 10102-18-8 |
